# Control of cardiovascular risk in black Africans with type 2 diabetes in Senegal

**DOI:** 10.5830/CVJA-2011-040

**Published:** 2012-06

**Authors:** NV Yaméogo, A Mbaye, M Ndour, LJ Kagambega, H Diomande, R Hakim, A Thiam, A Diallo, SN Diop, D Diagne, B Diack, A Kane

**Affiliations:** Service de cardiologie de l’Hôpital Général de Grand Yoff, Dakar, Senegal; Service de cardiologie de l’Hôpital Général de Grand Yoff, Dakar, Senegal; Service de cardiologie de l’Hôpital Général de Grand Yoff, Dakar, Senegal; Service de cardiologie de l’Hôpital Général de Grand Yoff, Dakar, Senegal; Service de cardiologie de l’Hôpital Général de Grand Yoff, Dakar, Senegal; Service de cardiologie de l’Hôpital Général de Grand Yoff, Dakar, Senegal; Service de cardiologie de l’Hôpital Général de Grand Yoff, Dakar, Senegal; Service de cardiologie de l’Hôpital Général de Grand Yoff, Dakar, Senegal; Service de cardiologie de l’Hôpital Général de Grand Yoff, Dakar, Senegal; Service de cardiologie de l’Hôpital Général de Grand Yoff, Dakar, Senegal; Service de cardiologie de l’Hôpital Général de Grand Yoff, Dakar, Senegal; Service de cardiologie de l’Hôpital Général de Grand Yoff, Dakar, Senegal

**Keywords:** diabetes, cardiovascular risk factors, control, diabète, facteurs de risque cardio-vasculaire, contrôle

## Abstract

**Introduction:**

Morbidity and mortality from diabetes are compounded by associated cardiovascular risk factors. For this reason, taking care of these risk factors is a public health goal.

In this study we evaluated the level of control of cardiovascular risk factors in black Africans with type 2 diabetes in Senegal.

**Methods:**

Between March 2007 and July 2008, we recruited type 2 diabetes patients from outpatient care in a specialised hospital in Senegal. Data were collected on a survey form designed for this purpose. An electrocardiogram and laboratory examinations were also performed. The level of control of diabetes and associated cardiovascular risk factors were assessed, as recommended by the American Diabetes Association (ADA).

**Results:**

A total of 318 type 2 diabetes subjects (237 women) were recruited. The average age was 58.2 ± 9.2 years (40–85). The mean duration of diabetes was 6.9 ± 5.9 years. The average glycaemic level was 1.4 ± 0.5 g/l and glycated haemoglobin was 7.6 ± 3.2%. The average length of patient follow up was 6.7 ± 6.1 years with a single annual consultation; 63.2% of the patients were on an insulin + biguanide combination, with good diabetes control (HbA_1c_ < 7%) in 25% of cases. Antihypertensive drugs were prescribed in 28.1% of hypertensive patients. More than half (51.9%) of these hypertensive patients were treated with angiotensin converting enzyme inhibitors. Their blood pressure was well controlled (< 130/85 mmHg) in 5.4% of the hypertensive patients (10/185). The low-density lipoprotein (LDL) cholesterol goal was achieved in 18.5% of cases (5/27).

**Conclusion:**

This study shows that the prevalence of cardiovascular risk factors is higher among black Africans suffering from type 2 diabetes. The control of these factors was not optimal in our study.

## Abstract (fr)

**Introduction:**

La morbidité et la mortalité du diabète sont aggravées par l’association de facteurs de risque cardiovasculaire dont la prise en charge est un objectif de santé publique. Nous évaluons dans cette étude le niveau de contrôle des facteurs de risque cardio-vasculaire dans une population de diabétiques de type 2 noirs africains au Sénégal.

**Méthodologie:**

Nous avons recruté, entre mars 2007 et juillet 2008, des diabétiques de type 2 suivis en ambulatoire en milieu hospitalier spécialisé au Sénégal. Les données ont été recueillies sur une fiche d’enquête conçue à cet effet. Un électrocardiogramme et des examens biologiques ont été aussi réalisés. Le niveau de contrôle du diabète et des facteurs de risque cardio-vasculaire associés a été évalué selon les recommandations de l’American Diabetes Association (ADA).

**Résultats:**

Un total de 318 diabétiques de type 2 (237 femmes) ont été colligés. L’âge moyen était de 58.2 ± 9.2 ans (40–85). La durée moyenne d’évolution du diabète était de 6.9 ± 5.9 ans avec un taux moyen de glycémie de 1.4 ± 0.5 g/l et d’hémoglobine glyquée de 7.6 ± 3.2%. La durée moyenne du suivi des patients était de 6.7 ± 6.1 ans avec une seule consultation annuelle; 63.2% des patients étaient sous l’association biguanide + insuline avec un bon contrôle du diabète (HbA_1c_ < 7%) dans 25% des cas. Les antihypertenseurs étaient prescrits chez 28.1% des hypertendus dont plus de la moitié (51.9%) sous inhibiteurs de l’enzyme de conversion de l’angiotensine avec un bon contrôle de la tension artérielle (< 130/85 mmHg) dans 5.4% des cas (10/185). L’objectif de LDL-cholestérol était atteint dans 18.5% des cas (5/27).

**Conclusion:**

Cette étude montre que la prévalence des facteurs de risque cardio-vasculaire est élevé chez les diabétiques de type 2 noirs africains. Le contrôle de ces facteurs n’était pas optimal dans notre étude.

## Résumé

Le diabète sucré est une maladie grave dont la prévalence est en constante progression. Il constitue un important facteur de risque cardio-vasculaire, avec une morbi-mortalité aggravée par les facteurs de risque communément associés telles que l’obésité, la dyslipidémie et l’hypertension artérielle.^1^ Il mérite d’être évalué dans ce sens surtout après une longue durée d’évolution.^2^ Il requiert un suivi continu et rigoureux des patients afin d’éviter la survenue les complications.^3^ La prise en charge des facteurs de risque cardio-vasculaire chez le diabétique de type 2 constitue ainsi un objectif prioritaire.^4^-^6^ Les objectifs de cette prise en charge sont de réduire l’incidence des complications dégénératives et le risque de survenue d’événements cardiovasculaires, d’augmenter l’espérance de vie et d’améliorer la qualité de vie de ces patients.

Notre étude avait pour but d’évaluer la prise en charge des facteurs de risque cardio-vasculaire et leur niveau de contrôle chez les diabétiques de type 2 au Sénégal.

## Patients et méthode

Nous avons mené une étude transversale, descriptive, multicentrique de Mars 2007 à Juillet 2008 dans trois des principaux centres hospitaliers de Dakar (Centre Hospitalier National Abass Ndao, Hôpital Général de Grand Yoff, CHU Aristide Le Dantec). Elle a porté sur les diabétiques de type 2 des deux sexes âgés d’au moins 40 ans.

Tous les patients ont bénéf icié d’un interrogatoire, d’un examen physique, d’un ECG et d’un bilan biologique comprenant la glycémie, l’hémoglobine glycosylée, la créatininémie, la microabuminurie, une protéinurie des 24 heures et un lipidogramme (cholestérol total, HDL-cholestérol, LDL-cholestérol, triglycérides).

Les facteurs de risque cardio-vasculaire modif iables recherchés étaient l’hypertension artérielle (HTA), la sédentarité, le tabagisme actif ou sevré depuis moins de 3 ans, l’obésité, la dyslipidémie et la microalbuminurie. Les atteintes des organes cibles recherchées étaient les atteintes cardio-vasculaire, rénale et cérébrale. Nous avons analysé le traitement en cours et considéré les objectifs thérapeutiques de l’American Diabetes Association (ADA).^7^-^9^ Ces objectifs comprenaient une pression artérielle < 130/85 mmHg, un taux de LDL-cholestérol < 1 mg/ dl et une hémoglobine glyquée < 7%.

Les données ont été recueillies à l’aide d’une fiche d’enquête, saisie sur micro-ordinateur et analysées à l’aide du logiciel SPSS version 17 for Windows.

## Résultats

L’échantillon était composé de 318 diabétiques de type 2 dont 237 femmes (74.5%) et 81 hommes (25.5%). L’âge moyen des patients était de 58.2 ± 9.2 ans (extrêmes : 40–85 ans). La durée moyenne d’évolution du diabète était de 6.9 ± 5.9 ans (extrêmes: 0.1–31 ans). La durée moyenne du suivi des patients était de 6.7 ± 6.1 ans (extrêmes : 0.1–28.5 ans) avec une seule consultation annuelle. La glycémie moyenne était de 1.4 ± 0.5 g/l (extrêmes : 0.7–4.9 g/l), celle de l’hémoglobine glycosylée était de 7.6 ± 3.2% (extrêmes: 4.5–10.9%) (tableau 1).

**Tableau 1.** Caractéristiques Générales Des Diabétiques De Type 2 (*n* = 318)

*Facteurs de risque*	*Population globale*	*Femmes*	*Hommes*
Sédentarité (%)	82.4	87.19	77.27
Hypertension artérielle (%)	58.2	58.2	58.0
Tabac (%)	6.0	3.8	17.3
Dyslipidémie (%)	43.1	36.3	62.9
Microalbuminurie (%)	36.8	30	25.9
Obésité (%)	10.1	12.6	7.8
Consommation d’alcool (%)	3.8	0.8	12.3

Les facteurs de risque cardio-vasculaire de nos patients sont indiqués dans le tableau I. Ils étaient dominés par la sédentarité (82.4%), l’hypertension artérielle (58.2%), la dyslipidémie (43.1%) et la microalbuminurie (36.8%).

Plus de la moitié des patients (63.2%) était sous l’association biguanide + insuline + mesures hygiéno-diététiques. Les différents traitements antidiabétiques utilisés figurent dans le tableau 2.

**Tableau 2.** Utilisation Des Traitements Antidiabetiques

*Traitement*	*Effectif (n)*	*Pourcentage (%)*
Régime seul	91	28.6
Biguanide + insuline	201	63.2
Biguanide	21	6.6
Sulfamides	5	1.6
Total	318	100

Les antihypertenseurs étaient prescrits chez 28.1% des hypertendus. Les inhibiteurs de l’enzyme de conversion de l’angiotensine étaient les médicaments les plus utilisés (51.9%). Les médicaments utilisés pour le traitement de l’hypertension artérielle figurent dans le tableau 3.

**Tableau 3.** Utilisation Des Medicaments Antihypertenseurs

*Médicaments antihypertenseurs*	*Effectifs (n)*	*Fréquence (%)*
Inhibiteurs de l’enzyme de conversion	27	51.9
Antagonistes des récepteurs de l’angiotensine II	8	15.4
Bêta bloquants	8	15.4
Diurétiques	12	23.1
Inhibiteurs calciques	5	9.6

L’aspirine était prescrite dans 6,9 % des cas. Quant aux statines, ils étaient prescrits chez 8.5% des patients.

Le diabète était contrôlé dans 14.8% des cas. Dans 75.2% des cas, l’hémoglobine glyquée était supérieure à 7%; elle était comprise entre 7 et 8% chez 38.4% des patients et supérieure à 8 chez 36.8% des diabétiques.

Parmi les patients hypertendus, 28.1% avaient un traitement antihypertenseur. La tension artérielle était supérieure à 130/85 mmHg chez tous les hypertendus non traités et chez 80.8% des hypertendus traités (soit 42 patients). L’hypertension artérielle était ainsi contrôlée dans 5.4% des cas. Lorsque le seuil de 140/90 mmHg était considéré, elle était contrôlée dans 11.9% des cas.

La dyslipidémie à LDL était observée dans 39.3 % des cas soit 125 patients. Parmi ces patients, 27 étaient sous statines. Une LDLémie normale était retrouvée chez 18.5 % d’entre eux. Dans la population globale, la LDLémie était inférieure à 1 g/l dans 62.3% des cas et inférieure à 1.3 g/l dans 67.9% des cas.

L’indice de masse corporelle était normal (< 25 kg/m^2^) dans 57.7% des cas. La surcharge pondérale était retrouvée dans 32.2% des cas et l’obésité dans 10.1% des cas.

La pratique d’une activité physique régulière n’était retrouvvée que chez 17.6% des diabétiques.

Le tabagisme était observé dans 6.0% des cas dont 2.5% avaient arrêté la consommation mais depuis moins de 3 ans.

## Discussion

Moins de 15 % de nos patients étaient bien contrôlés sur la base de l’hémoglobine glyquée. Moins du tiers des patients étaient contrôlés dans l’étude de Mcfarlane.^10^ Dans l’étude française ESPOIR, la proportion des patients contrôlés était de 27%.^11^ Ces résultats illustrent toute la difficulté de contrôle du diabète.

Très peu de patients étaient sous traitement antihypertenseur dans notre étude; seulement 28.1% d’entre eux. La valeur cible n’était obtenue que chez 5.4% des patients hypertendus. Dans l’étude ESPOIR,^11^ moins du tiers des patients étaient contrôlés sur le plan de la tension artérielle bien que la valeur cible fut 140/90 mmHg. La difficulté de contrôle de la pression artérielle serait liée aux comorbidités qui rendent difficile la prise en charge, mais aussi à l’inadéquation du traitement (peu sont traités).^10^ Ces observations confirment la nécessité d’utiliser souvent une polymédication antihypertensive pour atteindre la cible thérapeutique. Dans notre étude, seuls 13 patients étaient sous bithérapie antihypertensive et 6 sous trithérapie antihypertensive.

L’hypercholestérolémie à LDL était de 39.3% dans notre étude. La proportion des patients qui avaient une LDLémie inférieure à 1 g/l était de 62.3%. Dans l’étude de Mcfarlane,^10^ 35.5% des patients avaient un LDL cholestérol < 1 g/l. Dans l’étude ESPOIR, 66% des patients avaient une LDLémie < 1.3 g/l. Ce taux a été retrouvé dans 67.9% des cas dans notre étude. La prescription des statines n’était pas courante dans notre étude, et concernait seulement 8.5% des patients.

L’utilisation des antiagrégants plaquettaires était très faible dans notre étude (6.9%). Or selon les recommandations^12^ la prescription d’antiagrégant plaquettaire est justifiée chez les diabétiques qui ont en plus un des éléments suivants : cardiopathie ischémique, artériopathie des membres inférieurs, hypertension artérielle ou hypercholestérolémie. Cette situation était présente chez 52.51% de nos patients.

## Conclusion

Ce travail montre une prévalence élevée des facteurs de risque cardio-vasculaire modifiables chez les diabétiques de type 2 noirs africains. Le contrôle du diabète et des facteurs de risque associés n’était cependant pas optimal dans notre étude. Cette étude montre par ailleurs que la prise en charge des facteurs de risque cardio-vasculaire est souvent inadéquate chez les diabétiques noirs africains. Les recommandations internationales méritent d’être suivies pour une meilleure prise en charge de nos patients.
